# Shaping a suitable EU HTA dossier template: why the German template is not fit for purpose

**DOI:** 10.1007/s10198-023-01631-5

**Published:** 2023-10-16

**Authors:** Maria Katharina Schweitzer, Manuel Nico Dold, Astrid Genet, Klaus Gossens, Thomas Klein-Hessling, Nils Löffler, Matthias Rabel, Andrej Rasch, Eva-Maria Reuter, Jessica Schmelcher, Natalia Wolfram, Sebastian Werner

**Affiliations:** 1 AMS Advanced Medical Services GmbH, Am Exerzierplatz 2, 68167 Mannheim, Germany; 2 AMS Advanced Medical Services GmbH, Rosa-Bavarese-Str. 5, 80639 Munich, Germany; 3 Pfizer Deutschland GmbH, Linkstraße 10, 10785 Berlin, Germany; 4 AbbVie Deutschland, GmbH & Co. KG, Mainzer Straße 81, 65189 Wiesbaden, Germany; 5 Verband Forschender Arzneimittelhersteller e.V., Hausvogteiplatz 13, 10117 Berlin, Germany

**Keywords:** AMNOG, IQWiG, G-BA, EU HTA, PICO, JCA, I10, I18

## Abstract

From 2025, Health Technology Developers (HTDs) have to submit EU HTA dossiers. The joint clinical assessment (JCA) aims to streamline HTA processes and access to medicinal products across Europe. Currently, German HTA bodies IQWiG and G-BA actively shape the JCA methodology. Here we examine if German HTA dossier requirements are suitable for the JCA. We compare the number of safety endpoint and subgroup analyses in German dossiers with analyses considered in IQWIG’s benefit assessment and evaluate if these analyses were considered by the G-BA. We further investigated how the number of analyses was affected by the latest change in the German dossier template. With the current template, HTDs report in median 2.6 times more analyses on adverse events (AE) and 1.1 times more subgroup categories than in the previous template. IQWiG does not consider 33% of AE analyses and 73% of the subgroup categories presented by the HTD under the current template. G-BA considered the same AE as IQWiG in 76% of cases. Subgroups were uncommented by G-BA in most cases, independent of the template (previous: 93%, current 85%) and unconsidered in the conclusion on additional benefit (previous: 77%, current 69%). Thus, changes in the dossier template drastically increased HTD workload, but additional analyses seem unconsidered by the HTA bodies. With a broader scope in JCA, this effect could be amplified. To mitigate duplicative efforts and ensure prompt availability of medicinal products as envisioned by the HTAR, we suggest well-chosen and precise dossier requirements, early consultations, and early HTD engagement.

## Introduction

### EU health technology assessment: joint clinical assessments

While drug approval in Europe is usually a centralized process, reimbursement and pricing decisions are regulated at the national level. Consequently, different national health technology assessment (HTA) infrastructures exist across Europe resulting in different reimbursement decisions and access to innovative health technologies for patients. In an effort to harmonize the quality of evidence generation among European countries, reduce duplication of efforts for HTA bodies and industry [1] and improve the rapid and widespread availability of medicinal products across Europe, the Regulation 2021/2282 on health technology assessment (HTAR) entered into force in January 2022 and will be applied progressively [2].

As of January 2025, oncological drugs and advanced therapy medicinal products will have to undergo a joint clinical assessment (JCA). Orphan drugs will follow in 2028. In 2030, the HTAR will be fully applicable to all medicinal products and it will be mandatory for Health Technology Developers (HTDs) to submit clinical evidence on product benefit (EU HTA dossier) for the JCA [2]. In the market authorization application, the HTD answers the question whether a medicinal product is safe and effective, i.e., “does it work?”. By contrast, in the JCA dossier, the HTD answers the question whether the medicinal product is more effective than the standard of care, i.e., “is it better?”. Importantly, the European JCA will only focus on the patient-related clinical evaluation of the medicinal product against one or more comparators. An economic evaluation and the appraisal of the JCA will be conducted on a national level [3].

The HTAR forms the legal and organizational framework for the future cooperation on EU HTA. Its success will depend on a clear methodology as well as the practicability of the framework, requirements, and processes. Since 2010, these aspects were addressed by the European Network for Health Technology Assessment (EUnetHTA). Initially, the network collaboratively developed the EUnetHTA Core Model^®^, which is a standardized framework developed for HTAs across European countries. It provides a common structure for the clinical evaluation, safety, and economic evaluation of health technologies. The model covers both the assessment and appraisal phases of HTA [3]. However, following an EU decision in 2021, national authorities will be responsible for the appraisal component of EU HTA. Thus, the EUnetHTA Core Model^®^ in its current form is not applicable for the future JCA. As a result, the EU HTA template required adjustment to align with the revised approach that focusses exclusively on the assessment phase. In the latest EUnetHTA EU-wide voluntary project Joint Action 3, EU HTA procedural rules and methodology were drafted [4]. From 2021, on the base of Joint Action 3, processes, methods, and requirements for the EU HTA have been further developed by the EUnetHTA21 consortium [5]. The current work of the EUnetHTA21 consortium will be crucial for the successful implementation of the HTAR and represents an essential milestone for future European methods and processes in HTA.

The board of EUnetHTA21’s consortium is composed of 13 HTA bodies from 12 countries. Germany is the only country with two HTA bodies: The German Institute for Quality and Efficiency in Health Care (Institut für Qualität und Wirtschaftlichkeit im Gesundheitswesen, IQWiG) is a EUnetHTA founding member [6] whereas the German Federal Joint Committee (Gemeinsamer Bundesausschuss, G-BA) has become a full member of EUnetHTA in late 2015 [7]. In addition to the two German HTA bodies, agencies from the following countries are part of the EUnetHTA21 consortium: Spain, Italy, Austria, France, Portugal, Belgium, Ireland, Hungary, Norway, Sweden, and Netherlands [8]. In the course of the EUnetHTA21 project, the G-BA and/or the IQWiG contributed in hands-on groups and drafted guidance, such as: (1) scoping guideline, (2) methodological guideline for direct and indirect comparisons, (3) practical guideline for direct and indirect comparisons, (4) validity of clinical studies, (5) applicability of evidence, (6) submission dossier template, and (7) JCA report template [9–14]. Consequently, the perspective of the German HTA bodies and their HTA approaches plays a major role in the future EU HTA. The activities of the German HTA bodies have even intensified in recent years. The G-BA has increasingly taken lead and co-lead roles within Joint Actions 1–3. The IQWiG, while initially merely co-authoring and reviewing various guidelines, has subsequently been involved in many work packages and steering committees. In particular, the IQWiG engaged in quality assurance and standardization of operating procedures and processes [6]. The German HTA agencies will also play an important role in the future EU HTA coordination group that will continue the work of EUnetHTA21. The coordination group consists of the EUnetHTA21 members and HTA bodies from the remaining EU countries: Bulgaria, Croatia, Cyprus, Czech Republic, Denmark, Estonia, Finland, Greece, Latvia, Lithuania, Luxembourg, Malta, Poland, Romania, Slovakia, Slovenia, Liechtenstein, and Iceland [15].

### Timeline and scope of EU HTA processes

The future EU HTA process will run in parallel with the European Medicines Agency (EMA) drug approval process of a medicinal product and comprises three phases: the scoping phase, the dossier preparation/submission, and the assessment phase.

In the scoping phase, member states report their PICO questions (*P*opulation, *I*ntervention, *C*omparator, *O*utcomes) to be answered in the JCA in a so-called PICO survey. National needs and healthcare systems vary across Europe. According to the HTAR, the assessment scope for the JCA should be inclusive and reflect the needs of all member states in terms of data and analyses [1, 16]. The assessment scope is defined through consolidated PICOs that represent the framework for the definition of the research question. Thus, different comparators and outcomes might arise and the PICOs are anticipated to be strongly influenced by participating national HTA bodies. While the current EUnetHTA21 estimate is 4–6 PICOs, a recent analysis on the launch of a new lung cancer drug revealed that up to ten different PICOs can be expected within one submission [17]. While HTDs will need to submit available evidence to support such a large number of PICOs, the timeframe for dossier preparation will be very limited. The timelines have not yet been finally defined, but the previous analyses of possible timelines suggest very short durations for dossier preparation between scoping and submission [18], varying according to the type of approval procedure from 6.5 to 1.5 months (with average clock-stop durations). For a standard procedure, 6.5 months are expected. These numbers fall to 2.5 months and 15 days with minimal clock-stop durations. For accelerated marketing approval procedures, which are usual in the area of advanced therapy medicinal products and oncology drugs due to high unmet medical need, the maximum period may be reduced to 1.5 months. The most recent proposal by EUnetHTA21 suggests a fixed duration of approximately 2.5 months for dossier preparation regardless of the duration of the regulatory process.

Thus, EU HTA dossier submission must be completed within a very limited timeframe. It is essential to ensure that the submission is made no later than 45 days prior to the envisaged Committee for Medicinal Products for Human Use (CHMP) opinion, which is the basis for the European Commission's decision to grant or refuse a central marketing authorization [19]. During the assessment phase, the submitted EU HTA dossier is reviewed and follow-up requests are made to the HTD, if necessary. The JCA report will be endorsed by the coordination group 30 days after the European Commission's decision [1]. In a factual accuracy check (“fact check”) prior to endorsement, the HTD is given 5 days to review the formal correctness of figures and spelling. Thus, the involvement of the HTD on the final report of the JCA is limited to the reporting of purely technical or factual inaccuracies [20, 21]. The final JCA report will be considered for national appraisals and decision-making regarding pricing and reimbursement [1].

The EU HTA methodological requirements and its submission dossier template have not yet been finalized but will include all commonly used methods of evidence synthesis of Joint Actions 3 and EUnetHTA21 deliverables, including multiple sets of PICOs [22]. Considering the inclusive scope, there is a legitimate concern that extensive national HTA requirements on PICO, data, and analysis will be implemented in the assessment scope for the future EU HTA dossier. The information that is currently available from the draft submission dossier template and submission dossier guidance [23] strongly suggests that the German dossier requirements (based on the Act on the Reform of the Market for Medicinal Products (Arzneimittelmarktneuordnungsgesetz, AMNOG) might serve as a blueprint for the EU HTA dossier [24]. Furthermore, in 2021, the G-BA declared their efforts to ensure that the high and differentiating standards applying in Germany are also reflected in the European assessment [25, 26]. Therefore, it is conceivable that the German AMNOG requirements could be carried forward to the JCA and substantially influence the approach in the future EU HTA.

### HTA in Germany: the AMNOG process

Since 2011, HTDs in Germany are required to submit clinical data in the form of a dossier to the G-BA in accordance with the German AMNOG when they launch a new drug or are granted an additional marketing authorization [27]. To assess the additional benefit with regard to existing therapies, the medicinal product under evaluation is compared to a comparator defined by the G-BA. After dossier submission to the G-BA, the IQWiG is commissioned to conduct a benefit assessment based on this dossier and/or the available data of the underlying clinical study or studies. Following IQWiG’s benefit assessment, the G-BA decides on an additional benefit for the medicinal product, which is a decisive step for pricing in Germany [28]. Importantly, the G-BA is the only institution that can make a formal decision on the additional benefit, and thus the G-BA can overrule IQWiG’s recommendation [29]. This is not only a theoretical possibility, but differences between IQWiG’s recommendations and G-BA’s decisions sometimes occur [30–33].

The AMNOG benefit dossier consists of five modules that must be prepared according to dossier templates. Module 1 summarizes the dossier, Module 2 provides general information about the medicinal product, and Module 3 includes information on current therapy options, the target population, and estimated costs. Module 4 is a central component that contains the clinical data as the basis for the relative effectiveness assessment. Module 5 is a confidential annex with the underlying documentation, references, documentation of clinical trials, and authorization documents [22, 28, 34]. Procedural rules and dossier templates in the German AMNOG process place extensive requirements on the dossiers submitted and necessitate numerous statistical analyses.

Since the start in 2011, AMNOG requirements have been notably high in terms of analysis and documentation [35]. In April 2020, a revised AMNOG dossier template for Module 4 came into force [36], demanding the presentation of additional data cutoffs, additional analyses of efficacy endpoints, adverse events (AE) and additional subgroup (SG) analyses. This substantially expanded the required data analyses within Module 4, particularly impacting the demands on AE and SG with the mandatory presentation of SG analyses for age, gender, ethnicity/region, and severity of disease for all endpoints including safety broken down by SOC and PT [24]. This led to an increase of the average dossier size for Modules 1–4 by a factor 4 to 5, from an average of 750 to 3500 pages (in individual cases 20,000 to even 40,000 pages). The preparation of such dossiers requires enormous resources from HTDs, takes on average 12 months, and causes costs of 1,000,000 EUR [24].

### Aim and question

The objective of EU HTA is to minimize duplicative work for both HTA bodies and industry [1], and to improve the rapid and widespread availability of medicinal products across Europe [2]. For the success of EU HTA, EUnetHTA21 and the EU HTA coordination group have to establish a clear methodology and ensure that the framework, requirements, timelines, and processes are both feasible and practical. As part of both EUnetHTA21 and coordination group, the two German HTA bodies IQWiG and G-BA actively shape the future EU HTA and essentially contribute to finalize the requirements for the JCA. Here we asked: Are the current German AMNOG dossier requirements a suitable template for the future JCA given the objectives of the HTAR and the limited timeframe for dossier preparation? To answer this question, we focused on the sections of the dossier with the most extensive requirements (safety endpoints and SG) and compared information presented by HTDs in AMNOG dossiers, with the information that was assessed by the IQWiG and that was included in G-BA’s resolution on product benefit. First, we evaluated the number of safety endpoints and SG characteristics included in the AMNOG dossiers and IQWiG’s benefit assessment. We further qualitatively assessed whether the G-BA followed the IQWiG in terms of the selected AE in the resolution on product benefit and in terms of subgroup selection in the justification of the resolution. To underscore the significance of thoughtful dossier requirements and to highlight how a modification in these requirements can affect the scope of HTA dossiers, we further compared the number of safety endpoints and SG characteristics in the current assessments with assessments based on the previous dossier templates.

## Methods

Benefit dossiers submitted by the HTD to the G-BA, IQWiG’s assessments as well as resolutions and justifications of the G-BA were examined for endpoints on AE and SG characteristics. Assessment procedures based on both the previous and current dossier templates were selected. Only the data cutoff considered in the G-BA assessment was evaluated. Further cutoffs, required for all endpoints and SGs according to the template, were not included in our analysis.

### Data selection

Assessment procedures based on the current dossier template included all assessments with a start date after April 1, 2020 (i.e., IQWiG’s benefit assessments published between July 1, 2020 and June 15, 2021, G-BA’s resolutions and justifications from September 17, 2020 to September 15, 2021). Procedures based on the previous dossier template included assessments with start dates before April 1, 2020. To ensure comparability between current and previous dossiers, assessments submitted under the current dossier template were matched with their most recent counterparts under the previous template with similar therapeutic area and/or research question. If the most recent dossier under the previous template was not suitable (e.g., because it did not include study data), a suitable alternative was selected. For two current assessment procedures, matching previous assessment procedures could not be identified.

Only assessment procedures that covered the full spectrum of the dossier template requirements were considered. Others, e.g., orphan drug assessments or assessments based on single-arm studies, were excluded.

### Data extraction and quality assurance

The number of analyses on AE and SGs was extracted from the benefit dossiers of the HTDs and from IQWiG’s benefit assessments including addenda. Specifically, the number of AE differentiated by severity, broken down by System Organ Class (SOC) and Preferred Term (PT), and the number of adverse events of special interest were extracted from the dossiers. For IQWiG’s benefit assessments, AE were extracted from the results section and the tables in the appendices. Data of the mandatory SG characteristics, *age, sex, ethnicity/region* and *severity of disease*, as well as the number of any other SG characteristics presented, were recorded for HTD dossiers and IQWiG’s benefit assessments.

The IQWiG evaluates the HTA dossier and conducts the benefit assessment, the G-BA uses this assessment to draw a conclusion and to announce the formal decision. The G-BA does not perform the primary assessment. This also means that the G-BA usually does not add additional information to what has been selected by the IQWiG. Furthermore, the information published by the G-BA is usually more condensed and less detailed—for example, the G-BA does not list all the AE broken down by SOC and PT from IQWiG’s appendices; instead, they only focus on those mentioned in the results section. For these reasons, we quantitatively analyzed the number of analyses for HTD and the IQWiG, but qualitatively assessed if the G-BA followed the IQWiG in terms of the selected AE in the resolution on product benefit and in terms of subgroup selection in the justification of the resolution.

In addition to data on AE and SG, we extracted general information such as product name, date of the G-BA resolution, and template version. If the G-BA based its decision on a meta-analytic summary of several studies or an indirect comparison, only those were extracted, not individual studies. If multiple studies were presented but not meta-analyzed under the current template and only one study was presented in the matched assessment under the previous template, only the most similar study to the matched assessment in terms of design, patient numbers, etc., was extracted.

Quality control of the extraction was performed by a second, independent reviewer. Discrepancies were resolved jointly by both reviewers.

### Statistical analyses

The extracted data were analyzed descriptively. For categorical data, frequencies and percentages are reported. For continuous data, median, arithmetic mean, minimum, and maximum are presented. Analyses of the source documents from the different institutions (HTD, IQWiG, and G-BA) and from current and previous templates were performed independently.

The variables, *All endpoints on AE (incl. all SOC and PT)* and *Total number of SG characteristics*, describe the overall presentation effort for AE and for SGs for a benefit dossier. *All endpoints on AE* is a summation of the number of all *AE of any severity*, *serious adverse events*, *severe AE*, *non-severe AE*, *discontinuation due to AE* by SOC and PT, and all *non-severe*, *severe*, and *serious adverse events of special interest*. *Total number of SG characteristics* is a summation of all SG characteristics.

## Results

We compared the number of data analyses presented by HTDs under the current and the previous AMNOG dossier template with the proportion of analyses actually considered by the IQWiG in its benefit assessment (IQWiG’s perspective). We further qualitatively assessed if the G-BA followed the IQWiG in terms of the selected AE by comparing IQWiG’s results section with G-BA’s resolution on product benefit and assessed if the G-BA included the subgroups in its justification of the resolution (G-BA’s perspective).

### IQWiG's perspective

Out of 94 selected assessment procedures, 46 were based on the previous [34] and 48 on the current dossier template [36]. The therapeutic areas concerned were oncological diseases (47 procedures), diseases of the respiratory system (*n* = 19), the musculoskeletal system (*n* = 7), the nervous system (*n* = 3), psychiatric disorders (*n* = 2), cardiovascular diseases (*n* = 2), infectious diseases (*n* = 5), skin diseases (*n* = 4), metabolic diseases (*n* = 4), and ophthalmic diseases (*n* = 1), equally distributed between the previous and current dossier template.

Table 1 provides an overview of the number of endpoints on AE presented by HTDs and the IQWiG for the previous and the current dossier templates. Compared to the previous dossier template, the median number of reported data in the current template increased for almost every AE category, except for the categories *serious adverse events by SOC/PT* and *non-severe AE by SOC/PT*. The latter category was generally not considered by the IQWiG, although mandatory according to the dossier template.

**Table 1:** Overview of the endpoints on AE reported in benefit dossiers by HTDs and in IQWiG's benefit assessments

	Previous dossier template^a^			Current dossier template^b^		
Endpoint category	Reported by HTDs	Reported by the IQWiG	Difference in medians	Reported by HTDs	Reported by the IQWiG	Difference in medians
Number of EP						
Any AE by SOC/PT						
Median	24	27	− 3	59	49	10
Mean	74	40		109	70	
Min; max	(0; 705)	(0; 197)		(2; 1376)	(0; 718)	
Serious adverse events by SOC/PT						
Median	3	3	0	5	5	0
Mean	13	9		17	7	
Min; max	(0; 109)	(0; 107)		(0;382)	(0; 30)	
Severe AE by SOC/PT						
Median	2	0	2	15	2	13
Mean	14	7		30	10	
Min; max	(0; 101)	(0; 49)		(0; 574)	(0; 38)	
Non-severe AE by SOC/PT						
Median	0	0	0	0	0	0
Mean	39	0		14	0	
Min; max	(0; 859)	(0; 0)		(0; 91)	(0; 0)	
Discontinuation due to AE by SOC/PT						
Median	0	8	− 8	48	32	16
Mean	16	29		54	32	
Min; Max	(0; 126)	(0; 127)		(0; 246)	(0; 95)	
Adverse events of special interest						
Median	17	6	11	24	7	17
Mean	21	8		28	9	
Min; max	0; 90	0; 30		0; 144	0; 36	
All endpoints on AE (incl. all SOC and PT)						
Median	69	72	− 3	181	122	59
Mean	177	94		252	127	
Min; max	(8; 1801)	(0; 363)		(7; 2590)	(4; 822)	

AE: Adverse events; HTDs: Health Technology Developers; IQWiG: Institute for Quality and Efficiency in Health Care (Institut für Qualität und Wirtschaftlichkeit im Gesundheitswesen); PT: Preferred Term; SOC: System Organ Class

^a^Previous dossier template: according to resolution dated April 18, 2013

^b^Current dossier template: according to resolution dated February 21, 2019, effective April 2020

### Endpoints on adverse events

In dossiers using the current template, HTDs reported a median of 20% more evaluations than the IQWiG for *any AE by SOC/PT*. For *severe AE by SOC/PT*, the difference was at median 87%. A maximum of 101 *severe AE by SOC/PT* were reported in a dossier with the previous template. With the current template, the maximum number of *severe AE by SOC/PT* was 574. The IQWiG, on the other hand, never considered more than 50 *severe AE by SOC/PT* under either the previous or current dossier templates. In the category *discontinuation due to AE by SOC/PT*, the IQWiG considered in median only two thirds of the evaluations presented by HTDs. A very large difference appeared for *adverse events of special interest*, where the IQWiG reported less than one third of the analyses submitted by HTDs. When analyzing *all endpoints on AE*, including *all SOC and PT*, the number of analyses reported by HTDs was almost three times higher with the current dossier template than with the previous template. In contrast, the IQWiG reported only 69% more of *all endpoints on AE* with the current template, that is 67% of the median number of evaluations presented by HTDs in this category. The range of reported AE also illustrates the discrepancy between the two parties: the maximum number of reported endpoints for the category *all endpoints on AE including all SOC and PT* amounted to 2590, while the IQWiG never considered more than 822.

### Subgroups

The German dossier templates mandate the presentation of SG analyses for *age, gender, ethnicity/region* and *severity of disease* across all endpoints, including safety data broken down by SOC and PT to investigate potential effect modifications. More than 95% of the evaluated dossiers complied with these requirements.

Table 2 shows the *total number of SG characteristics* reported. The median number of SGs presented by HTDs slightly increased with the current template, while this number decreased for the IQWiG. The IQWiG reported only 27% of the SG analyses presented by HTDs under the current template. This difference in extent of presented SG analyses was also reflected in the maximum number of SGs reported: 24 for HTDs and only 6 for the IQWiG.

**Table 2:** Overall overview of reported results on SG analyses in benefit dossiers by HTDs and IQWiG's benefit assessments

	Previous dossier template^a^			Current dossier template^b^		
	Reported by HTDs	Reported by the IQWiG	Difference in medians	Reported by HTDs	Reported by the IQWiG	Difference in medians
Total number of SG characteristics						
Median	10	4	6	11	3	8
Mean	10	4		11	3	
Min; max	(0; 22)	(0; 8)		(0; 24)	(0; 6)	

HTDs: Health Technology Developers; IQWiG: Institute for Quality and Efficiency in Health Care (Institut für Qualität und Wirtschaftlichkeit im Gesundheitswesen); SG: subgroup

^a^Previous dossier template: according to resolution dated April 18, 2013;

^b^Current dossier template: according to resolution dated February 21, 2019, effective April 2020

Under the current template, the SG *age* was considered in 81% of IQWiG's benefit assessments. The SG *gender* was considered in 73% of the assessments. With the previous template, the SG *ethnicity/region* was considered in 61% of the assessments. This number dropped to 23% with the current template, meaning that three quarters of the analyses presented for this SG were not considered by the IQWiG. The SG *severity of disease* was considered by the IQWiG in less than half of its assessments (48%) based on the current template.

Apart from the standard SGs listed above, HTDs are required to report a priori planned SG analyses as defined per study protocol for all patient-relevant endpoints [37]. Our analysis revealed that the IQWiG almost never considered any other SGs than the standard SGs. This applies to both the previous and current dossier templates. In individual cases of other SG characteristics, assessments of disease-specific SGs were considered.

### G-BA’s perspective

Following IQWiG’s benefit assessment, the G-BA issues a resolution to define the additional benefit of the medicinal product and explains its reasoning in more detail in a justification [28]. Our analyses of IQWiG’ s results section and G-BA’s resolution show that the G-BA considered the same AEs as the IQWiG in 76% of the cases, under both the current and previous templates.

Single AEs that show a statistically significant effect are occasionally considered to support the evaluation and derivation of the additional benefit. With the introduction of the current dossier template, the proportion of these supportively reported AEs slightly increased (from 43 to 56%).

IQWiG's selection of the SGs *age, sex, ethnicity/region,* and *severity of disease* was adopted by the G-BA in at least half of the assessment procedures based on either the previous or current templates and remained uncommented in most of the cases (93% vs. 85%). SG analyses were, however, mostly unconsidered in the conclusion of the G-BA on the additional benefit (72%). SG analyses for safety endpoints, especially those by SOC/PT, were never considered in the final G-BA decision on additional benefit. Differences between resolutions based on the previous and current templates were minor (76% vs. 69%). In 18% of the cases, an evaluation of the data was not possible, because the relevant data were missing in the publicly available sections of the HTD’s dossier.

## Discussion

Currently, HTA requirements differ vastly between European countries [38–41]. In comparison to other HTA submissions requirements, the German AMNOG system seems to be particularly demanding [42, 43]. The G-BA has released an updated dossier template in 2020, which imposes even more rigorous requirements. As a result, HTDs are now expected to provide substantially more analyses. In particular, AE and SG analyses were affected by this change. Here we show that the average number of AE analyses almost tripled under the current as compared to the previous template.

The requirements of the current template were logically expected to also inflate the evaluations of the dossiers performed by the IQWiG and the G-BA. Our analysis, however, reveals a large discrepancy between the number of AE and SG analyses that are submitted by HTDs in accordance with the requirements and those evaluated in the benefit assessments by the IQWiG. With a larger data set and with the comparison between the previous and current dossier templates, our findings corroborate and extend an earlier analysis [22].

Although the G-BA occasionally reports a few statistically significant AE to support its final decision on the additional benefit, some of the mandatory AE, such as *non-severe AE* for instance, are never considered, and *any AE by SOC/PT* are rarely taken into account.

The G-BA's procedural rules request SG analyses for *gender, age, severity of disease or stage,* and *country and study center effects* to investigate potential effect modifications [28]. When applicable, SGs for a priori defined potential effect modifiers should also be reported. The SG analyses should be performed for all EP, even though G-BA’s decisions on the additional benefit seldom rely on SG analyses. SG analyses for safety endpoints, especially those by *SOC/PT*, representing the majority of the SG analyses, were never considered in the final decision on additional benefit. The number of SGs must also be considered in the context of the increased numbers of endpoints, for which SG analyses have to be presented. Even a modest increase in the number of SGs is potentiated by an enhanced number of endpoints, which can lead to a major increase in the total numbers of required SG analyses. In our analysis, we focused on the data cutoff that was considered in the G-BA assessment. Nevertheless, according to the dossier template, the HTD is required to provide additional data cutoffs for all EPs and SGs, which remained unconsidered. Incorporating these additional data cutoffs into our analysis, however, would have resulted in a further multiplication of our findings.

### Implications for the JCA

We are convinced that the success of EU HTA will strongly depend on the clarity and workability of its assessment framework, particularly the dossier requirements. Despite the high-quality standards of the German AMNOG dossier templates in terms of the amount of data published, this study suggests that a critical review of the German dossier template and a reduction of the requested analyses is crucial, if the German template is intended to serve as a blueprint for the JCA. We substantiate previous findings that most of the extensive submission requirements of the German dossier template are unnecessary [24]. The current German dossiers are inflated by many analyses that require a substantial investment of time but are irrelevant in IQWiG’s assessment and for G-BA’s decision on additional benefit. Thus, an unfiltered inclusion of the national German requirements in JCA dossiers would merely export unnecessary analyses to the European level.

German dossier templates are, along with many other documents related to the AMNOG process, publicly accessible. However, there is a lack of transparency regarding the selection methods employed by the IQWiG for its benefit assessment. As a result, HTDs cannot comprehend precisely which submitted data the IQWiG utilizes in its assessment, further compounded by the absence of comments. This is consistent with criticisms of the methodology's lack of clarity and transparency, even after Joint Action 3. A further learning from Joint Action 3 is that the adopted methodology should strive for consistency across assessments. These aspects highlight the crucial need for IQWiG and G-BA to provide clearer descriptions and justifications for their assessment methods utilized. Doing so could aid in reducing the workload for both parties [44].

According to the HTAR, the JCA’s assessment scope “shall be inclusive and reflect member states’ needs in terms of parameters and of the information, data, analyses and other evidence to be submitted by the HTD” [1], particularly the PICOs. EUnetHTA21’s final scoping proposal considered limiting the PICOs to a maximum of five in complex cases for new medicinal products [16]. However, the PICO approach—in which the combined and consolidated PICO sets of the member states influence each other—proposed for some indications could result in a much larger number of PICOs. A recently published evidence-based study showed that ten PICOs may be required for the indication of lung cancer [17]. The number of analyses will further increase with the application of the requirements (outcomes and SGs) to each single PICO. Thereby, safety analyses might represent the largest number of additional analyses if the current German requirements are included in the consolidated PICO sets. According to the current requirements of the German HTA process, the aforementioned analysis for lung cancer indicated that 430 safety analyses had to be submitted for 10 PICOs. This clearly shows that the German AMNOG dossier has particularly high requirements for safety analyses with the mandatory presentation of SG analyses for age, gender, ethnicity/region, and severity of disease across all endpoints, including safety data broken down by SOC and PT. An (early) HTA is conducted at the time of authorization, where it is statistically and epistemologically feasible to identify only preliminary and coarse safety signals [45, 46], which contrasts sharply with a robust and extensive safety evaluation at this level of detail (SOC and PT). Further, our analysis and other findings clearly highlight that most of these extensive safety data in the German dossier are not being considered [22]. Consequently, safety analyses for the future JCA dossier should not align with the exceptionally high requirements for safety analyses from the German dossier templates. However, if these requirements were to be adopted by the JCA for all relevant PICOs, it would disproportionately increase the amount of additional safety information required, while at the same time disregard up to 70% of the safety analyses for the additional benefit assessment and appraisal [17]. This accumulation would surpass the current demand for analyses across Europe, which clearly contradicts the initial objective of the HTAR to reduce duplication.

In addition, the unnecessary inflation of EU HTA dossiers by analyses irrelevant to most of the member states would result in dossiers that do not fully meet the needs of any HTA body while augmenting the requested amount of work for both HTDs and the institutions. Such extensive and unnecessary data requests even jeopardize the efficacy of the joint EU HTA. A reasonable number of consolidated PICOs for each JCA is crucial to ensure the feasibility of EU HTA. The member states must, therefore, agree on a core set of PICOs (“European PICOs”) relevant to the majority, with a focus on populations and comparators. The definition of a common set of outcomes (endpoints, AE, SGs) for a given indication or agent is also encouraged instead of the currently anticipated inclusive scope. The rapid and broad availability of medicinal products throughout Europe relies on a thorough definition of the mandatory analyses. It is now the responsibility of the European Commission and the member states to develop a practical European submission template for JCAs [22] while taking into account the open issues from Joint Action 3 and learnings from different studies [17, 24]. Ideally this template should undergo regular evaluation and be improved accordingly.

### Further recommendations for the EU HTA

This analysis highlights that the current German AMNOG dossier requirements are not a suitable template for the EU HTA dossier and should not be adopted. The JCA should reflect the common needs of the member states as reflected by 1. the EMA requirements, 2. the scientific consensus, 3. the patient’s perspective, and 4. the available evidence. The early definition of the needs of the European member states via public PICO surveys, ideally in coordination with the EMA processes, would enable their implementation already in pivotal studies.

To facilitate the incorporation of PICOs that are of joint interest to the EU member states in the study planning, it is imperative to meaningfully engage HTDs from an early time. Consequently, mandatory and early joint scientific consultations available for each procedure should be included. While European multi-HTA Early Dialogues were regularly held during Joint Action 3 [44], in the current version of the EU HTA process, they are no longer included. This is particularly unfortunate given the clear learnings from Joint Action 3 that early consultation meetings to discuss methodology and the PICO structure would be beneficial. These meetings could allow for a more informed and efficient preparation of the dossier [45]. The engagement of HTDs is limited to the preparation of the dossier and a fact checking during the assessment phase. Joint scientific consultations are planned with HTA bodies and EMA only with limited capacities and only if specific selection criteria are met (e.g., drug is first-in-class in the respective indication) [1]. HTDs currently have no legal right to attend a joint scientific consultation and must apply for limited offers [46]. When not offered a consultation, HTDs would have to approach each national HTA body separately to clarify the HTA requirements for the study design and analyses of the data. This runs against the core objective of EU HTA to avoid duplications of work at the national level and facilitate HTA assessments. We, therefore, strongly recommend involving HTDs in the scoping process. In addition, a mandatory joint scientific consultation with the HTD should be enabled for every single medicinal product subjected to a JCA and provide clear, reliable, and timely guidance on the required clinical evidence [47]. Scoping meetings should be held before the start of the pivotal clinical trial(s) and give advice on trial design (e.g., in terms of comparators, *endpoints*, and health outcomes) [48] to ensure that the generated clinical evidence will be valuable for both regulatory and EU HTA purposes. This would not only save the resources of all parties involved but also lead to better studies, resulting in improved HTA outcomes. This, in turn, would enhance patient access to innovations.

A timely and high-quality output can only be achieved if the EU HTA process truly delivers a joint assessment output (one “JCA”) that focuses on common aspects of the member states’ health systems, rather than trying to meet all their individual needs. The JCA should prioritize the development of common European methodologies, information requirements, and harmonized HTA criteria, instead of attempting to merge various national practices. Under current guidelines, national HTA bodies could request additional evidence from the HTDs if justifiable (see HTAR legislative act I §15) [1]. Therefore, there is no need to request outcomes only relevant to one member state into the European submission. However, considering the findings from current research and given that many of the German requirements seem to be even questionable for the German HTA procedure, we do not encourage additional data requests at the national level, on top of the JCA. In summary, member states should carefully decide on the required evidence for the JCA and reduce it to the mere necessary. Lean and precise requirements targeted to the indication and product are essential, especially given the short timelines of the EU HTA.

In addition, an adjustment of the proposed timelines is warranted. The latest proposal regarding the EU HTA timelines from EUnetHTA21 suggests a fixed duration of approximately 2.5 months for dossier preparation irrespective of the duration of the regulatory process, while it takes an average of 12 months to compile the German AMNOG dossier. Nevertheless, HTDs face major challenges to meet the German submission timelines. Should the EU HTA requirements be as, or even more, extensive than the current German template, meeting the compressed EU HTA timelines will not be feasible [22]. Therefore, it seems crucial to amend the HTAR, even if it requires an Implementing Act, to allow for sufficient time for dossier preparation. This would also allow for the synchronicity of marketing authorization and EU HTA. Without these significant adjustments, the suggested timeframe might put the timely finalization of the EU HTA dossier at risk and delay access to new medications for European patients.

### Methodological considerations

This study only considered the German AMNOG process. Other national HTA processes may, however, have requirements that would further augment the volume of the EU HTA dossier. While the AMNOG process remains highly pertinent, given the leading role of the German institutions in EUnetHTA21 and the future coordination group, it would be interesting to incorporate the perspective of other European countries in future analyses and discussions.

Our analysis focused on AE and SG, because these aspects were strongly affected by the changes introduced in the current dossier template for Module 4. In addition, as compared to safety endpoints, there are generally less efficacy endpoints in AMNOG dossiers and only very few are considered as patient relevant by the G-BA. Importantly, however, all endpoint categories in the AMNOG dossier (mortality, morbidity, quality of life, safety) can be influenced by the change because an additional subgroup or an additional data cutoff affects all endpoints. Also note that we only focused on the data cut evaluated by the G-BA; however, sometimes HTDs present more than one data cutoff according to the dossier template requirements. This can further exacerbate the discrepancy between presented and evaluated analyses.

At last, we wish to acknowledge that the discussion section might be biased toward HTDs’ point of view. This bias cannot be overcome as neither the IQWiG nor the G-BA provide a public statement explaining why they do not consider large amounts of analyses. We can only assume that both IQWIG and G-BA ignore large parts of the presented analyses, because they do not regard them as relevant for the benefit assessment. Furthermore, because HTDs are a key stakeholder in HTA, there are only few publications on this matter that are not coauthored by an industry partner.

## Conclusion

In conclusion, the current German AMNOG dossier requirements are not a suitable template for the EU HTA dossier. The German dossier template is very demanding and requests HTDs to present many analyses, in particular for safety endpoints and subgroups. Yet, most of these analyses are not considered in the assessment process. Thus, the requirements need to be reassessed and reduced to ensure a more efficient process. Using the current version of the German requirements as a template for the EU HTA would export unnecessary analyses to the European level. Extensive AMNOG dossiers necessitate lengthy preparation times, spanning approximately 12 months [22], which is incompatible with the proposed EU HTA timeframe of 2.5 months. As a result of these divergent timeframes, there is a substantial risk that patient accessibility could face delays. For a functional and efficient EU HTA, only necessary analyses should be requested. Furthermore, it is imperative to include mandatory and early joint scientific consultations to meaningfully engage HTDs from an early time. An early engagement of HTDs would also facilitate the incorporation of PICOs that are of joint interest to the EU member states in the study planning. In our view, thoughtfully selected dossier requirements, mandatory early joint scientific consultations, and HTD engagement would reduce the duplication of efforts for HTA bodies and industry and ensure rapid and widespread availability of medicinal products across Europe, as envisaged by the HTAR.

## Data Availability

The data generated and analyzed in the analysis are included in the article.
